# Calpain Cleavage of Brain Glutamic Acid Decarboxylase 65 Is Pathological and Impairs GABA Neurotransmission

**DOI:** 10.1371/journal.pone.0033002

**Published:** 2012-03-12

**Authors:** Chandana Buddhala, Marjorie Suarez, Jigar Modi, Howard Prentice, Zhiyuan Ma, Rui Tao, Jang Yen Wu

**Affiliations:** Department of Biomedical Science, Charles E Schmidt College of Medicine, Florida Atlantic University, Boca Raton, Florida, United States of America,; University of Illinois-Chicago, United States of America

## Abstract

Previously, we have shown that the GABA synthesizing enzyme, L-glutamic acid decarboxylase 65 (GAD65) is cleaved to form its truncated form (tGAD65) which is 2–3 times more active than the full length form (fGAD65). The enzyme responsible for cleavage was later identified as calpain. Calpain is known to cleave its substrates either under a transient physiological stimulus or upon a sustained pathological insult. However, the precise role of calpain cleavage of fGAD65 is poorly understood. In this communication, we examined the cleavage of fGAD65 under diverse pathological conditions including rats under ischemia/reperfusion insult as well as rat brain synaptosomes and primary neuronal cultures subjected to excessive stimulation with high concentration of KCl. We have shown that the formation of tGAD65 progressively increases with increasing stimulus concentration both in rat brain synaptosomes and primary rat embryo cultures. More importantly, direct cleavage of synaptic vesicle - associated fGAD65 by calpain was demonstrated and the resulting tGAD65 bearing the active site of the enzyme was detached from the synaptic vesicles. Vesicular GABA transport of the newly synthesized GABA was found to be reduced in calpain treated SVs. Furthermore, we also observed that the levels of tGAD65 in the focal cerebral ischemic rat brain tissue increased corresponding to the elevation of local glutamate as indicated by microdialysis. Moreover, the levels of tGAD65 was also proportional to the degree of cell death when the primary neuronal cultures were exposed to high KCl. Based on these observations, we conclude that calpain-mediated cleavage of fGAD65 is pathological, presumably due to decrease in the activity of synaptic vesicle - associated fGAD65 resulting in a decrease in the GABA synthesis - packaging coupling process leading to reduced GABA neurotransmission.

## Introduction

The brain constantly strives to maintain a balance between the excitatory and inhibitory networks, whose key players are the neurotransmitters L-glutamic acid and γ-amino butyric acid (GABA) respectively. The decision in maintaining the state of equilibrium is controlled by the activity of the enzyme L-glutamic acid decarboxylase (GAD; EC 4.1.1.15), which is responsible for the synthesis of GABA from the substrate L-glutamic acid [1]. Too much excitation or too little inhibition could tip the balance, potentially triggering a plethora of neurodegenerative diseases such as epilepsy, general anxiety disorder, schizophrenia, Parkinson's disease, Huntington's chorea, etc. all of which have varied etiologies but harbor a fundamental disruption of the local GABA circuitry [2]–[8]. Synaptic inhibition by GABA is not only confined to the fine tuning of inhibitory neurotransmission in the central nervous system, but also encompasses the peripheral nervous system where it is actively involved as an endocrine signaling molecule [1]. GABA inhibition failure due to GAD auto-immunity has gained widespread attention and is implicated in diseases such as insulin-dependent diabetes milletus (IDDM), Stiff person syndrome, Batten disease, bi-polar disorder, cerebellar ataxia etc [9]–[13].

Interestingly, in the mammalian brain, unlike any other neurotransmitter synthesizing enzymes, GABA is the only neurotransmitter featuring two catalytic enzymes, namely GAD65 and GAD67, where 65 and 67 denote their respective molecular weights in KDa [14]. While 90% of GABA is the outcome of constitutive expression by GAD67 in the cytosol channeled for non-neurotransmission functions, synaptic vesicle (SV) membrane - associated GAD65 is transiently activated to reinforce GABA for high frequency bursts to fine-tune GABAergic neurotransmission at the synapse [15]–[16]. In our earlier work, we demonstrated that there exists a functional coupling between vesicular GABA transporter (VGAT) and SV - associated GAD65 [17]. Such an alignment in close proximity between these two molecules, licenses VGAT to preferentially load the newly synthesized GABA generated by the transiently activated membrane - associated GAD65 into the SVs, over the pre-existing GABA constitutively generated by GAD67 in the soma. This key phenomenon is disrupted in GAD65^−/−^ mice. Mice deficient in GAD65 appear normal but are highly susceptible to epilepsy and general anxiety disorder which could be spontaneously precipitated by fear or mild stress [18]–[19]. These findings underscore the pivotal role played by membrane - associated GAD65 to regulate GABA for extra-synaptic tonic inhibition.

In our previous publications, we reported the presence of truncated GAD65 (tGAD65) during protein purification of human recombinant full length GAD65 (fGAD65) from a bacterial expression system *in vitro* [20]. fGAD65 could be readily cleaved by mild trypsin treatment or incubation with Factor Xa to yield a 58 KDa tGAD65, confirming an earlier purported report that a proteolytic hot-spot is contained within the first 100 amino acids of GAD65 [21]. Peptide fragment analysis revealed the identity of the cleavage site to be precisely between Arg69 and Lys70 [20]. Interestingly, *in vitro* biochemical characterization of fGAD65 versus tGAD65 indicated that the truncated form was 2–3 times more stable and stronger than the full length form [20]. In addition, we demonstrated that rat brain fGAD65 could be cleaved upon neuronal stimulation *in vivo* to yield a cleaved product, which had similar electrophoretic mobility as the purified recombinant tGAD65 on SDS-PAGE [22]. Furthermore, by various biochemical approaches, we demonstrated that the enzyme calpain was responsible for the cleavage of fGAD65 to form tGAD65 [22]. However, the conditions favoring calpain cleavage of fGAD65 and the implications it has on GABA neurotransmission have not been fully characterized.

In the CNS, calpain is a ubiquitous calcium sensitive cysteine rich protease that is involved in normal physiological processes and also appears to play a key role in neuropathological events. While transient calpain activation is necessary for numerous cell signaling and remodeling events, altered calcium homeostasis triggers an irrepressible calpain activation that causes irreversible neuronal damage [23]. Calpain cleavage of fGAD65 has two functional consequences. From the propensity of our previously published *in vitro* data, calpain cleavage of fGAD65 is physiological if the cleavage of fGAD65 to tGAD65 facilitates the production of a more active truncated form that could meet the extra demand for GABA. On the other hand, if the cleaved product containing the active site of the enzyme is no longer attached to SVs, this signifies a loss of function model leading to impairment of GABA synthesis-transport coupling process with VGAT that dampens neuronal firing resulting in a severe lapse in GABA neurotransmission.

The report presented here aims to understand the conditions that led to cleavage of fGAD65 and to elucidate how the outcome of the cleavage process regulates GABA neurotransmission. Our data indicated that the formation of tGAD65 progressed with an increase in pathological stimulus in both rat brain synaptosomes and primary rat embryo neuronal cultures. At the transcriptional level, fGAD65 mRNA was down-regulated with an increase in high K^+^ concentration. Furthermore, we demonstrated that fGAD65 on the SVs was amenable to cleavage by calpain and the site of cleavage was not masked by protein-protein interactions. Post cleavage, the active tGAD65 was liberated from the SVs. Interestingly, tGAD65 was observed in the transient focal cerebral ischemic rat brain tissue and not in the matched control regions further strengthening our notion that the formation of tGAD65 occurs under pathological conditions. Using a range of biochemical approaches, the functional significance of fGAD65 cleavage was also investigated. The data indicated that the cleavage resulted in reduced GABA synthesis and uptake at the SVs. This is the first communication addressing cleavage of fGAD65 directly on the SVs, the site of action of fGAD65, where the synthesis of GABA occurs specifically for the purposes of neurotransmission. We also conceptualized experiments that would serve as a proof of concept to our fGAD65 cleavage hypothesis. A prototype model integrating calpain cleavage of fGAD65 in regulating GABA neurotransmission is also discussed.

## Results

### 1. Effect of high K^+^ stimulation of rat brain synaptosomes on cleavage of fGAD65

**Figure 1:**
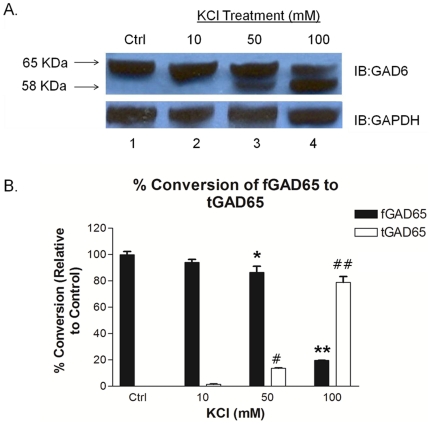
Effect of high K^+^ stimulation of rat brain synaptosomes on cleavage of fGAD65. **A.** Representative immuno-blot image showing progression in cleavage of fGAD65 to form tGAD65 when rat brain synaptosomes were stimulated with increasing K^+^ concentration (lanes 2–4). A non-stimulated synaptosomal fraction (lane 1) served as a control. The samples were analyzed by immuno-blotting using monoclonal GAD65-specific antibody; GAD6. The 58 KDa cleaved product tGAD65 was found to accumulate proportional to the increase in KCl concentration. **B.** Densitometric analyses of the conversion of fGAD65 to tGAD65 in rat brain synaptosomes treated with increasing K^+^ concentration expressed as % of control (Fig. 1B, Ctrl). Data are presented as mean ± SEM, n = 4 independent experiments. *P<0.05 and *^#^P*<0.05 are statistically significant differences between the levels of fGAD65 and tGAD65 of 50 mM KCl treatment group and the control respectively. **P<0.001 and *^##^P*<0.001 are statistically significant differences in the levels of fGAD65 and tGAD65 between 100 mM KCl treatment group and the control respectively.

When fresh rat brain synaptosomes suspended in Kreb's Ringer Phosphate (KRP) buffer were stimulated with increasing concentrations of K^+^, progressive accumulation of the 58 KDa product; tGAD65 in a dose dependent manner was observed (Fig. 1A, lanes 3 and 4). The presence of the cleaved product was demonstrated by immunoblotting using a monoclonal antibody GAD6, whose epitopes are directed against the C terminus of the protein (see methods). tGAD65 was essentially absent at physiologically low concentrations of K^+^ (10 mM K^+^; Fig. 1A, lane 2) and in the control sample (Fig. 1A, lane 1), respectively. However, upon exposure to higher concentrations of K^+^, tGAD65 was more readily released.

### 2. Effect of high K^+^ stimulation of rat brain synaptosomes on total GAD65 activity

**Figure 2:**
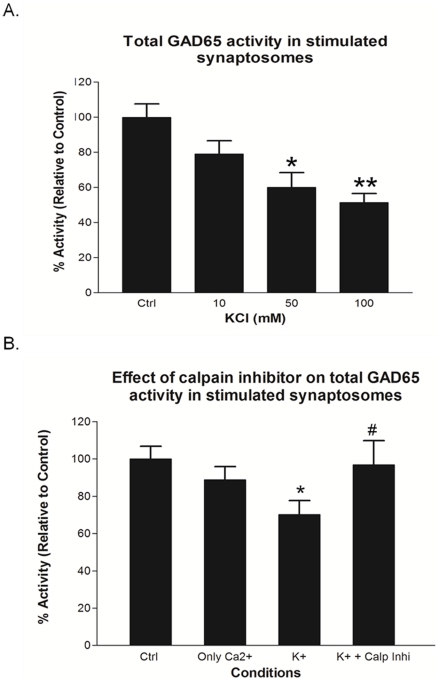
Effect of high K^+^ stimulation of rat brain synaptosomes on total GAD65 activity. **A.** Total GAD65 activity in the rat brain synaptosomes was measured under conditions promoting cleavage of fGAD65. Synaptosomes were stimulated with increasing concentrations of K^+^ as indicated (Fig. 2A; 10 mM KCl group, 50 mM KCl group and 100 mM KCl group). Unstimulated synaptosomes served as the control (Fig. 2A; Ctrl group). Total GAD65 activity comprising the activity of both fGAD65 and tGAD65 after stimulation was measured using a combinational approach of immunoprecipitation and radiometric GAD activity assays. First, soon after stimulation, total GAD65 was pulled down by immunoprecipitation using monoclonal GAD65 antibody; GAD6. Next, after normalizing the synaptosomal lysates between groups, the pulled down complex was analyzed using the classical radiometric method for measuring GAD activity. The values are mean ± SEM (n = 4); *P<0.05 and **P<0.005 are statistically significant differences between 50 mM KCl and 100 mM KCl treatment groups with the control group respectively. **B.** Effect of calpain inhibitor on total GAD65 activity was evaluated by pre-incubating the synaptosomes with 1 µM of calpain inhibitor peptide as shown in Fig. 2B. As in Fig. 2A, the synaptosomes in Fig. 2B, were subjected to 50 mM KCl treatment with or without the calpain inhibitor, followed by synaptosomal lysis, normalization, immunoprecipitation and then the total GAD65 activity was measured by radiometric GAD activity assays. Data are represented as ± mean SEM (n = 3); *P<0.05 are the statistically significant differences between K^+^ (50 mM) and the control groups. *^#^P*<0.05 are the statistically significant differences between K^+^ (50 mM) and the K^+^+Calp Inhi (50 mM KCl and 1 µM calpain inhibitor) groups.

Since neuronal stimulation promoted cleavage of fGAD65 to form tGAD65, we further wanted to evaluate the effect of stimulation of rat brain synaptosomes on total GAD65 activity as shown in Fig. 2. Briefly, after synaptosomal stimulation, the lysates from each treatment group served as a starting material for immunoprecipitation using monoclonal GAD65 antibody; GAD6, which pulled down both fGAD65 as well as the cleaved product; tGAD65. Classical radiometric GAD activity assay was performed on the immunoprecipitants. As shown in Fig. 2A, total GAD65 activity decreased with increase in the concentration of K^+^. A very high concentration of K^+^ at 100 mM, where maximal conversion of fGAD65 to tGAD65 occurred (Fig. 1A, lane 4) also sharply reduced total GAD65 activity to about 50% relative to the control (Fig. 2A, column 100 mM). Incubation with 1 µM calpain inhibitor peptide (Fig. 2B) restored total GAD65 activity to control levels indicating that inhibition of calpain has direct relation to activity.

### 3. Time dependent cleavage of fGAD65 and corresponding cell viability in rat primary neuronal cultures upon exposure to 100 mM K^+^ stimulation

**Figure 3:**
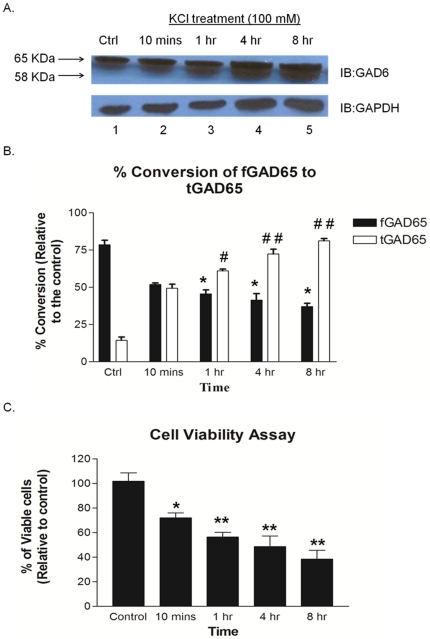
Time dependent cleavage of fGAD65 and corresponding cell viability upon exposure to 100 mM K^+^ stimulation in primary rat neuronal cultures. E17 primary rat neuronal cell cultures at 11 DIV were exposed to 100 mM KCl for different time intervals as shown. **A.** Representative immuno-blot analysis of fGAD65 cleavage post 100 mM KCl treatment (Fig. 3A, lanes 2–5). Lane 1 represents the untreated control. **B.** Quantitative analysis of fGAD65 cleavage in primary rat neuronal cells expressed as % of control is shown in Fig. 3B. Values are mean of ± SEM (n = 3); *P<0.001 is the statistically significant differences between the levels of expression of fGAD65 in 1 hr, 4 hr and 8 hr treatment groups and the control group respectively; *^##^P*<0.005 and *^#^P*<0.05 are statistically significant differences between the levels of expression of tGAD65 in the 1 hr, 4 hr and 8 hr treatment groups with the control group respectively. **C.** Cell viability of rat primary neuronal cells in culture under similar conditions as in Expt. 3A, which promoted fGAD65 cleavage. Values are mean of ± SEM (n = 4); *P<0.05 is the statistically significant difference between 10 min exposure and the control group. *P<0.001 is the statistically significant differences between 1 hr, 4 hr and 8 hr treatment with the control group respectively.

To further assess whether fGAD65 cleavage is observed in whole intact neuronal cells, rat primary neuronal cells in culture at 11 days *in vitro* (DIV) were subjected to 100 mM KCl treatment for different time points as indicated (Fig. 3A, lanes 2–5). tGAD65 was readily released and substantial aggregation of the cleaved product occurred at 4 hr and 8 hr post KCl treatment (Fig. 3A, lanes 4 and 5 respectively). Similar results were obtained when primary rat neuronal cultures were exposed to hydrogen peroxide or glutamate (unpublished data).

To further clarify whether the formation of tGAD65 occurs under pathological conditions, corresponding time dependent cell viability assay was performed using Promega Cell Viability Assay under the same conditions which promoted fGAD65 cleavage in primary neuronal cell cultures as shown in Fig. 3B. The data indicated that the conditions at which cleavage of fGAD65 occurred; also promoted neuronal cell death, demonstrating, that fGAD65 is cleaved under pathological conditions. At 4 hr of exposure to 100 mM KCl, about 50% of fGAD65 was cleaved to form tGAD65 (Fig. 3A, lane 4), around which, 50% loss of cell death occurred (Fig. 3B, lane 4 hr treatment).

### 4. Time dependent differential expression of GAD65 and GAD67 mRNA upon exposure to 100 mM KCl in primary rat neuronal cell cultures

**Figure 4:**
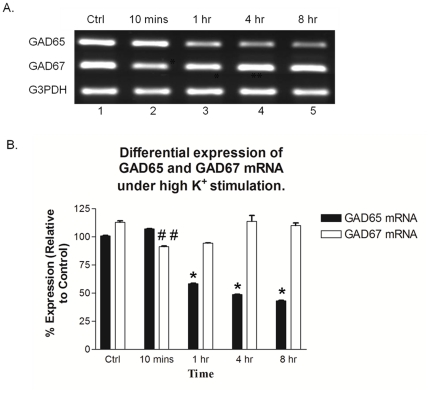
Time dependent differential expression of GAD65 and GAD67 mRNA upon exposure to 100 mM KCl in primary rat neuronal cell cultures. A. Representative gel image of GAD65 and GAD67 mRNA separated on a 1.5% agarose gel. Lane 1: Control; Lanes 2–5: 10 min, 1 hr, 4 hr and 8 hr exposure to 100 mM KCl respectively. GAD65 and GAD67 mRNA were differentially regulated under the same conditions of exposure to 100 mM KCl for different time points as indicated in lanes 2–5. B. Quantitative analyses of GAD65 and GAD67 mRNA expression shown as % of control (Fig. 4B, Ctrl). Values are mean of ± SEM (n = 4); *^##^P*<0.05 statistically significant differences between GAD67 mRNA expression of 10 min exposure group and the control. *P<0.05 statistically significant differences between GAD65 mRNA expression of 1 hr and 4 hr exposure groups with the control respectively. **P<0.005 is the statistically significant difference between GAD65 mRNA expression between 8 hr exposure group and the control.

Next, we evaluated the changes in the expressions of GAD65 and GAD67 mRNA under the same conditions as Expt #3, to determine how the GAD mRNA are regulated at the transcriptional level in response to the established pathological conditions that promoted the cleavage of fGAD65 to form tGAD65 (Expt #3). The mRNA levels of both GAD67 and GAD65 were differentially expressed. As shown in Fig. 4A, GAD65 mRNA expression was down- regulated with increase in exposure to 100 mM KCl in a time dependent manner. However, GAD67 mRNA expression initially decreased at 10 min exposure, and was up-regulated upon longer exposure time.

### 5. Cleavage of SV-associated fGAD65 as a function of KCl concentration

**Figure 5:**
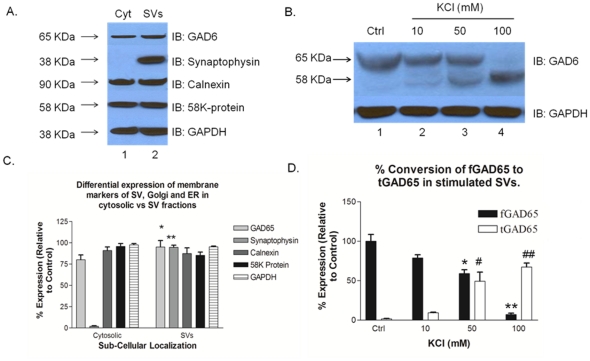
Cleavage of SV-associated fGAD65 as a function of KCl concentration: **A.** Representative immuno-blot demonstrating the isolation of enriched SVs, relatively free from any contaminating cytosolic fractions, other than that of Golgi and ER fractions, as indicated by immuno-blotting against synaptosophysin (SV marker), calnexin (ER marker) and 58K Protein (Golgi marker). **B.** Representative immuno-blot demonstrating cleavage of SV-associated fGAD65 directly on the SVs, on account of a dose-dependent KCl stimulation. **C.** Quantitative densitometric comparison, expressed as relative percentage with respect to SVs, of the levels of expression of fGAD65 and synaptophysin in SVs vs Cyt (cytosolic) fractions. Values are mean of ± SEM (n = 3). *P<0.05 and **P<0.001 are statistically significant differences in the levels of expression between fGAD65 and synaptophysin in the SV fractions when compared tocyt fractions. **D.** Densitometric analyses of the changes in the expressions of fGAD65 and tGAD65 in the SV fractions. Untreated SVs served as a control. Data points are mean of ± SEM (n = 3). *P<0.05 and *^#^P*<0.05 are statistically significant differences between 50 mM KCl treatment group and the control group respectively. **P<0.005 and ##P<0.005 are statistically significant differences between 100 mM KCl group and the control group.

It is well established that fGAD65 is found abundantly as a membrane protein tightly anchored to the SV membrane not through direct interactions, but by forming a protein complex with several proteins, including HSC70 and VGAT [17], [24]. Because SV-associated fGAD65 is directly responsible for neurotransmitter GABA, it therefore, became imperative to address whether the calpain cleavage site of fGAD65 on the SVs was susceptible to attack by the protease. To test this idea, we stimulated synaptosomes with increasing concentrations of KCl, after which the SVs were isolated and washed. These SVs were relatively free of contamination, except for ER and Golgi (see Fig. 5A) to separate them from any contaminating cytosolic fractions, as indicated by a representative immuno-blotting against synaptophysin ab, a SV marker (Fig. 5A). Golgi and ER are not involved in uptake of GABA since there is no evidence of VGAT in Golgi or ER. Enriched SVs were isolated and the cleaved fGAD65 was detected by immuno-blotting against GAD6 ab (Fig. 5B). As reported, we confirmed that the membrane-bound fGAD65 on the SVs could be readily cleaved to form tGAD65, similar to the cytosolic synaptosomal fractions (Fig. 1A). At 100 mM KCl stimulation, complete conversion of fGAD65 to tGAD65 was observed.

### 6. Demonstration of the release of tGAD65 from SV membranes - *In vitro* calpain cleavage assay

**Figure 6:**
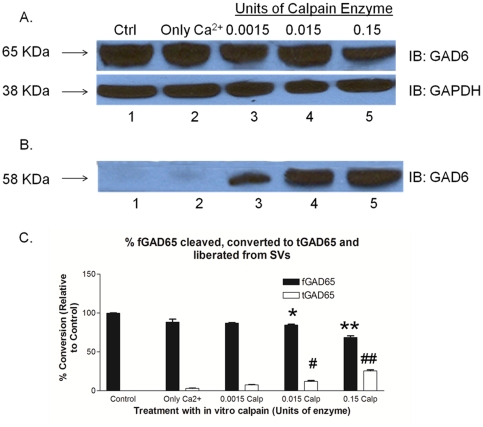
Status of tGAD65 on intact SVs that were subjected to *in vitro* calpain treatment. **A and B.** Representative immuno-blot image of liberation of tGAD65 when intact SV membranes were subjected to *in vitro* calpain cleavage. **A.** Washed intact synaptic vesicles **B.** Liberated tGAD65 fragments collected in the aqueous fraction. As indicated, both lanes 1 and 2 in Fig. 6A and Fig. 6B served as controls. Lanes 3–5 in Fig. 6A represent SV membranes that were washed after *in vitro* calpain treatment. Lanes 3–5 in Fig. 6B represent the tGAD65 fragments liberated into the aqueous fraction. **C.** Densitometric analyses of liberation of tGAD65 fragments. Values are mean ± SEM (n = 3); **P<0.005 and ^#*#*^ *P*<0.005 are statistically significant differences between fGAD65 and tGAD65 in the 0.15 calpain treatment with the untreated control respectively. *P<0.05 and ^#^ *P*<0.05 are statistically significant differences between fGAD65 and tGAD65 in the 0.015 calpain treatment with the untreated control respectively.

We further examined to confirm whether tGAD65 after calpain cleavage was still attached to the SV membranes. In order to test this idea, we designed an *in vitro* calpain cleavage assay to promote calpain cleavage of fGAD65 directly on the SVs. After calpain treatment, the SVs were centrifuged to separate the aqueous fraction from the SV membranes. Thereafter, the washed SV membrane fractions and the aqueous fractions were normalized, re-suspended in gel loading dye for further analysis. As shown in Fig. 6A, tGAD65 was no longer attached to SVs, but was found in the collected aqueous fraction (Fig. 6B).

### 7. Effect of calpain-mediated cleavage of SVs on GABA synthesis

**Figure 7:**
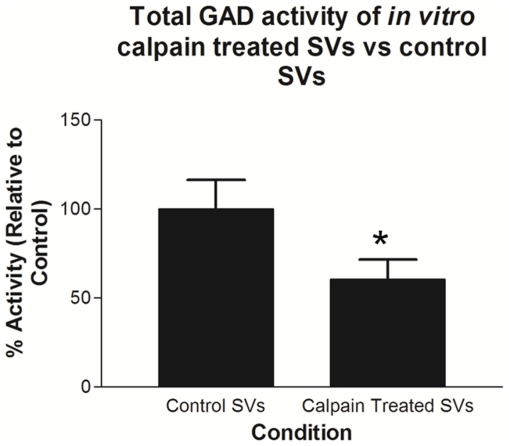
Effect of calpain-mediated cleavage of SVs on GABA synthesis. Residual GAD activity of calpain-treated and untreated SVs. Lane 1: Control SVs; Lane 2: SVs treated with 0.15 U of calpain enzyme. Data are mean of ± SEM (n = 3). *P<0.005 is the statistically significant difference in the GAD activity between the calpain-treated SV group and the control.

Since SV-associated fGAD65 is solely involved in synthesizing neurotransmitter GABA, we next investigated the effect of calpain-mediated cleavage of SVs on GABA synthesis. If tGAD65 detached from the SVs and liberated into the cytosol post calpain cleavage, as suggested by our data in Fig. 6A, one would expect to see a decrease in the synthesis of GABA in calpain-treated SVs. To test this concept, intact SVs were isolated and subjected to calpain treatment (0.15 U) *in vitro*. The control as well as calpain-treated SVs were washed and the residual GAD activity remaining on the SVs was assessed using the standard radiometric GAD activity assay as described [25]–[26]. As anticipated, calpain-treated SVs showed remarkable decrease in GABA synthesis of about 50% when compared to the control (Fig. 7).

### 8. Effect of calpain treatment of GABAergic SVs on newly synthesized GABA uptake

**Figure 8:**
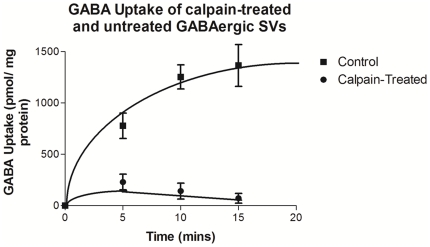
Effect of calpain treatment of GABAergic SVs on newly synthesized GABA uptake. Uptake of newly synthesized GABA was conducted in the presence (•) or absence (▪) of calpain enzyme. [^3^H] Glu was used as a substrate for the synthesis of newly synthesized [^3^H] GABA. Values are mean of ± SEM (n = 3). The specific uptake was obtained by subtracting the non-specific uptake from the total uptake and was expressed as pmol per mg of protein.

From our previously published data, it is well established that fGAD65 and VGAT on the SV membranes are functionally coupled to each other and that such a tight coupling between the two molecules ensures efficient GABA synthesis and packaging into the SVs [17], [27]. We then began to question how the uptake of newly synthesized GABA would be affected if tGAD65 was liberated from the SVs after calpain treatment. To examine this idea, we performed an experiment to monitor the radiometric newly synthesized GABA uptake profiles of both calpain-treated and control GABAergic SVs. Our data as shown in Fig. 8 reveal that the slope of the curve of calpain-treated GABAergic SVs was lower when compared to that of control, further strengthening our hypothesis that because of liberation of tGAD65 after cleavage, the coupling between VGAT and fGAD65 remaining on the SVs was compromised.

### 9. Identification of tGAD65 fragments in a rat model of focal cerebral ischemia and corresponding elevation of glutamate levels during ischemia

**Figure 9:**
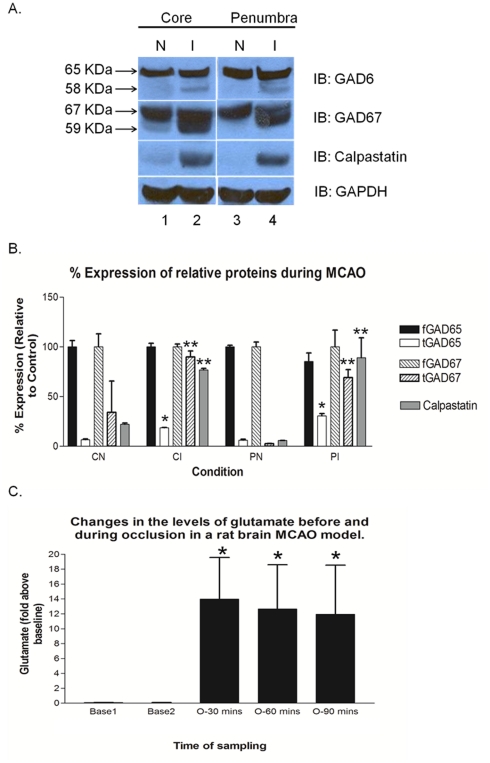
Identification of truncated forms of GAD and corresponding elevation of glutamate levels in a MCAO model. **A.** Representative immuno-blot demonstrating the presence of truncated forms of GAD65 and GAD67 and corresponding elevation of calpastatin. N, Normal; I, Ischemic. **B.** Densitometric analysis of the immuno-blot images. Data points are mean of ± SEM (n = 3). **P<0.005 and *P<0.05 are statistically significant differences when compared to their respective controls. **C.** Corresponding changes in the levels of glutamate before and during ischemia. Values are mean of ± SEM (n = 4). *P<0.005 is the statistically significant differences between during occlusion and before occlusion.

To show a proof of concept to our cleavage hypothesis of fGAD65, we envisioned to research for the presence of tGAD65 fragments in a rat model of focal cerebral ischemia. In order to carry out this investigation, a rat model of Middle Cerebral Artery Occlusion (MCAO) was created as described [28]. After 90 min of occlusion, brain tissue from the core and penumbra of the ischemic side and the matched control side were isolated, processed and immuno-blotted against GAD65 and GAD67 antibodies as shown in Fig. 9A. Interestingly, we observed that both fGAD65 and fGAD67 were cleaved to form their respective truncated forms. Apart from the GADs, we also assessed the changes in the levels of calpastatin, an endogenous calpain inhibitor using calpastatin antibody (Fig. 9A). We observed that the levels of calpastatin in the ischemic tissue of both core and penumbra were highly up-regulated when compared to the very low expression in the controls.

In a parallel analysis within the same animal before harvesting the brain tissue, we also monitored the corresponding changes in the extracellular glutamate release in the rat brain before and during occlusion by microdialysis, followed by HPLC. Our data indicate that there was a 20 fold rise in the glutamate levels during occlusion when compared to the baseline glutamate levels before occlusion (Fig. 9B). The rise in the levels of glutamate directly demonstrates that cleavage of fGAD65 and fGAD67 occurred under excitotoxic conditions in a rat stroke model.

## Discussion

There is ambiguity concerning whether cleavage of fGAD65 mediated by calpain is a physiological response or a pathological outcome, which has direct implications on GABA neurotransmission at the synapse [22], [29]. Recent reports suggest that cleavage of fGAD65 occurs under glutamate alterations that contribute to sustained calpain activation [30]–[31]. In this study, we have concluded that cleavage of fGAD65 reduced GABA neurotransmission, which is a pathological outcome of the cleavage mechanism. This is based on the following principal findings. Firstly, the accumulation of tGAD65 occurred under conditions of progressively increasing excitotoxic insult in rat brain synaptosomes. In rat primary embryo cell cultures, the factors which caused the formation of tGAD65 not only down-regulated GAD65 mRNA expression, but also promoted neuronal death, indicating that tGAD65 was formed during extreme pathological conditions. Secondly, total GAD65 activity, attributed to both fGAD65 and tGAD65 combined, was sharply reduced at a very high K^+^ concentration where the maximal cleavage of fGAD65 occurred. The total GAD65 activity was restored by the addition of calpain inhibitor prior to stimulation, suggesting a direct role of calpain in the cleavage of fGAD65 which affects activity. Thirdly, fGAD65 on the SVs, which is not directly attached to SVs but in association with interacting partners such as HSC70, CSP and VGAT [17], [24] on the SVs was amenable to calpain cleavage, suggesting that the calpain cleavage site of fGAD65 on the SVs was not masked by protein-protein interactions. Fourthly, after calpain cleavage, the active tGAD65 was no longer attached to the SVs but was liberated into the surrounding aqueous fraction. Importantly, total GAD activity as well as newly synthesized GABA uptake profiles of calpain-treated GABAergic SVs showed a reduction in total GAD activity and uptake respectively, when compared to the untreated control. Finally, in a rat model of transient, focal cerebral ischemia (MCAO), tGAD65 was observed in the core and penumbra of the ischemic tissue which showed corresponding elevation in glutamate release during occlusion, but no tGAD65 was detected in the matched controls, further strengthening our notion that formation of tGAD65 occurs under pathological conditions. Overall, the present findings suggest that the calpain cleavage of fGAD65 has deleterious functional consequences on the magnitude and sustenance of rapid firing required for the fine tuning of GABAergic synaptic function. Any perturbation to this norm can be directly linked to debilitating neurodegenerative diseases such as epilepsy, Parkinson's disease, and Huntington's chorea etc [32].

It is well known that the GABA generated by GAD65 and GAD67 are compartmentalized into synaptic and metabolic pools respectively [14]. GAD65 and GAD67 are both synthesized as soluble proteins, but only GAD65 undergoes exclusive post-translational modifications at the N terminus necessary for post-Golgi trafficking that targets the molecule to the SV membranes, its primary site of action [33]. No such modifications are known to occur for GAD67. The GAD molecules are highly homologous but exhibit small but extremely divergent sequence variation at the N terminus which dictate their affinities to co-factor pyridoxal L'-phosphate (PLP) [16]. The recently resolved crystal structure of the N-terminal truncations of both GAD isoforms reveal that GAD67 has a tethered loop covering the active site that boosts continuous GABA production, while the same catalytic loop is inherently mobile in GAD65, acting as a molecular switch, regulating the transient activation of GAD65 [34]. Because of these differences in the interaction with PLP, GAD67 is responsible for 90% GABA in the brain that is used for basal firing. On the other hand, GAD65 is transiently activated to augment GABA production necessary for sustained inhibitory bursts to the post-synaptic nerve terminal [16]. It has been documented that GAD65 and GAD67 both undergo homo or heterodimerization at the middle and the C terminus and that the removal of N terminus did not affect their activity [35]. Earlier views in the literature unanimously proposed that only GAD65, either as a homo or heterodimer attached to the SVs via protein-protein interactions [17], [24]. However, emerging data in understanding the membrane anchoring properties of GAD65 and GAD67 have revealed a robust GAD65-independent membrane anchoring, axonal targeting and pre-synaptic clustering of GAD67 on the SVs [36]. The attachment was abolished by a leucine-103 to proline mutation which changed the conformation of the N terminal domain but does not affect the GAD65-dependent membrane anchoring of GAD67 [37].

Applying this substantial body of knowledge regarding differential regulation and localization of the GAD molecules to fGAD65 cleavage, a number of important conclusions could be drawn. From our previously published peptide fragment analysis data, it was identified that the calpain-mediated cleavage of fGAD65 occurred between Arg 69 and Lys 70 [20]. Calpain-mediated cleavage of fGAD67 resulted in two shorter forms of tGAD67, occurring as a consequence of independent cleavages after amino acids 70 and 90 [38]. Thus, the calpain-mediated cleavage sites of the GAD molecules are lodged in their respective N termini. Incidentally, it has long been established that the residues participating in the attachment to SVs for GAD65 are resident in the N terminus of the molecule [39]. It has always been accepted that only GAD65 and not GAD67 attached to the SVs via partnership through various other molecules comprising the SV machinery [16]–[17]. Any availability of GAD67 at the SVs was speculated to be available through heterodimerization with GAD65 at the middle and the C terminus, where the functional residues of both the enzymes are resident [34]. However, recent data definitively demonstrated a robust GAD65-independent anchoring of GAD67 to SVs, and that the attachment was completely abolished by a leucine-103 to proline mutation, confirming that the N termini residues of GAD67 are also involved in the independent anchoring mechanism. Nevertheless, this single amino acid change had no effect on the GAD65-GAD67 heterodimerization [36]–[37]. Therefore, based on the above discussion, it is reasonable to propose that since the calpain-mediated cleavage of either of GAD65 or GAD67 in their homo/heterodimer states occurs at their corresponding N termini, the effect would be to liberate the functional part of the enzymes which no longer are proximal to VGAT, thereby disrupting the functional coupling process. This loss of function was further confirmed by a decline in the total GAD activity as well as new GABA uptake profiles in calpain-cleaved GABAergic SVs data presented in this investigation, suggesting that that the cleavage of fGAD65 enzyme has deleterious functional consequences. Furthermore, from another perspective, it became essential to shed light on the fate of GABA neurotransmission devoid of GAD65 and address any possible role played by cytosolic GAD67 in such a scenario. Studies on GAD65^−/−^ mice indicate that there is a decrease in the refilling kinetics of the releasable pool of vesicles under sustained synaptic activation but not under a spontaneous stimulus, suggesting that cytosolic GAD67-derived GABA could not substitute for the loss of GAD65-derived GABA required during high-frequency neuronal firing [40]. In an independent observation, from our vesicular uptake assays published earlier, it was evident that VGAT preferred the newly synthesized GABA synthesized by GAD65 over the pre-existing GABA synthesized by GAD67 [17]. The converse was true with GAD65^−/−^ mice [27]. In the course of the study, based on our data, we began to understand that fGAD65 cleavage is not only an outcome of a pathological process, but also that the cleavage caused reduced GABA uptake, with repercussions affecting the GABA release at the synaptic cleft, suggesting that calpain-mediated cleavage of fGAD65 is both a cause and effect phenomenon.

Though calpain activity has been implicated in normal physiological functions, altered calcium homeostasis leads to persistent, pathological activation of calpain [23]. Aberrant calpain activity is a hallmark of a variety of neurodegenerative diseases such as cerebral ischemia, Alzheimer's disease, Parkinson's disease, Huntington's disease, multiple sclerosis and amyotrophic lateral sclerosis [41]. Under high calcium influx, important neuronal proteins, specifically those comprising the SV machinery, negatively affect neurotransmitter release [42]–[44]. Calpain has long been identified as a potential therapeutic target and a myriad of cell-permeable calpain inhibitors have been synthesized for pharmacological inhibition of calpain activity [45]–[46]. Detection of tGAD65 fragments in our MCAO model of ischemia in the core and penumbra on the ipsilateral side of the brain and its absence in the matched contralateral side is a direct proof of concept to the cleavage hypothesis of fGAD65 that calpain-mediated cleavage of the GAD enzyme occurs under pathological conditions. Also, it is interesting to note that the sustained calpain activation that led to fGAD65 cleavage occurred in conjunction with a 20-fold increase in glutamate levels when compared to baseline, and that at such high levels of glutamate, the turnover rate of calpastatin, an endogenous calpain inhibitor, showed a drastic elevation in the injured tissue when compared to the control, perhaps as a response to rescue of the injured tissue. Although calpain is shown to be responsible for fGAD65 cleavage, the role of synchronous activity of calpain and caspases cannot be under estimated [47]–[49]. This is especially true from our primary neuronal cell culture data where the conditions which favored calpain cleavage of fGAD65 also promoted neuronal cell death. The commitment steps to the execution of apoptosis are initially directed by the calpain/calpastatin ratio, which is a deciding factor to trigger the activation of the caspase cascade [48], [50]. In addition to this, it is intriguing to note that it has been published elsewhere that there is decreased expression and activity of VGAT in rat brain following focal ischemia [51]. Therefore, it is reasonable to speculate that under sustained calpain activation such as in ischemia, calpain-cleavage of fGAD65, not only liberated the functional moiety of the enzyme from the site of action at the SVs causing a sharp decline in the refilling kinetics, but also knocked down VGAT expression and activity, thereby collectively depleting the readily releasable pool of GABA loaded SVs, perturbing the already established functional coupling process from our previous work [17]. Also, it has been shown earlier that GABA uptake into SVs by VGAT depends on the integrity of electrochemical gradients of SVs [52]. Under sustained stimulation such as the case discussed here, the local excesses of calcium in the pre-synaptic nerve terminal compromise the action of V-ATPases leading to severe loss of SV membrane integrity [53]–[54].

**Figure 10:**
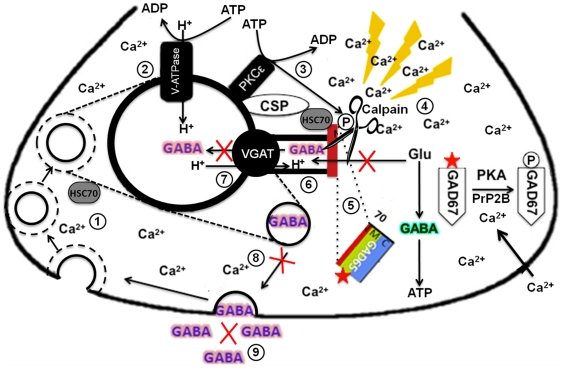
Proposed model of the SV-associated fGAD65 cleavage mediated by calpain under sustained neuronal stimulation. Under sustained neuronal stimulation, SV-associated fGAD65 which is in association with VGAT via partnership with HSC70 and CSP is cleaved by hyper-active calpain to liberate the functional part of the enzyme. This resulted in the disruption of the fGAD65-VGAT functional coupling process, largely reducing the releasable pool of GABA loaded SVs. The GABA generated by SV-associated fGAD65 is shown in magenta; GABA generated by fGAD67 is shown in teal. The stars on GAD67 and GAD65 represent the active states of the enzymes. GAD67 is activated by dephosphorylation, whereas GAD65 is active in its phosphorylated state. Certain areas of the pathway which are directly affected by calpain-mediated cleavage of fGAD65 are shown in red crosses. The sequence of events leading to liberation of tGAD65 is discussed in the text.

In conclusion, the deleterious functional consequences culminating from sustained calpain activation, following acute neuronal insult are depicted in the prototype model as shown (Fig. 10). The model is an amendment of our previously published model of fGAD65 interacting partners at the SVs [17]. The localization of the various molecules in a typical GABAergic neuron is presented. The transiently activated SV-associated fGAD65 (here shown cleaved), cross-talks with VGAT via its interacting partners, namely HSC70 and CSP and is involved in synthesizing new GABA neurotransmitter [16]–[17]. Free cytosolic fGAD67 in its de-phosphorylated state, constitutively synthesizes GABA for non-neurotransmission purposes to meet the high energy requirements in the neuron [16], [55]. Briefly, the changes occurring in the over-excited neuron are delineated as follows. 1. The SVs are recycled by means of clathrin-coated pits. The clathrin coat is then dissociated from the SVs through interaction with HSC70 [24]. 2. SVs are then returned to their resting state, where the proton gradient is restored by V-ATPase. 3. SV-associated fGAD65 is activated through protein phosphorylation by a proton gradient dependent protein kinase. We have shown *in vitro* that fGAD65 is phosphorylated by PKC€ [55]. In a typical neuron under a physiological stimulus, the activated fGAD65 uses glutamate from the cytosol to synthesize new GABA to be transported into VGAT 4. However, upon sustained stimulation, Ca^2+^ from intracellular stores is released into the cytosol, raising the intracellular calcium concentration, leading to hyperactivation of calpain. 5. Hyperactivate calpain translocates from the cytosol to act on its substrates, one of which is fGAD65. SV-associated fGAD65 is cleaved at the N terminus, thereby liberating the active part of the enzyme which is resident in the middle region of the molecule. 6. Since this is a loss of function at site model, active fGAD65 post cleavage is unavailable to generate newly synthesized GABA to be transported into VGAT. 7. VGAT is unable to take up any GABA owing to loss of the electrochemical gradient necessary to exchange protons for newly synthesized GABA. 8 and 9. The net loss is a reduction in the number of new GABA loaded SVs that are to be dispatched to the pre-synaptic nerve terminal, necessary to be released at the synaptic cleft.

## Materials and Methods

### Experiments involving animals

The protocol for this study for use of animals and their materials were approved by and in accordance with the requirements of Institutional Animal Care and Use Committee, Florida Atlantic University. The Public Health Service animal welfare assurance number is A3883-01. For all experiments, either 250–300 g Sprague-Dawley male rats (Harlan, IL, USA) or timed-mated Sprague Dawley pregnant female rats (Charles River, MA, USA) were used. The animals were separated by sex and two animals per cage were housed and maintained at 22°C with an alternating 12-hr light/dark cycle. The animals were allowed a minimum stabilization period of at least 3 days after which they were utilized for experimentation.

### 1. Isolation of synaptosomes

Crude synaptosomes were prepared as described previously [56]. Unless otherwise specified, all steps were performed with ice cold solutions. Rats were euthanized in a closed chamber with a lethal inhalation dose of isoflurane, following which they were decapitated using a guillotine. Brains were quickly isolated, suspended in Hepes buffered sucrose solution (0.32 M sucrose, 4 mM Hepes-NaOH, pH 7.4, 10 ml/g of brain w/v) and homogenized with the aid of Potter-Elvehjem Teflon dounce homogenizer. The homogenate was centrifuged at 1500 g for 10 min to remove the nuclear debris. The resulting supernatant (S1) was saved while the pellet (P1) was further homogenized in the same volume of sucrose buffer used in the previous step and was re-centrifuged under the same conditions to yield supernatant (S1') and pellet (P1'). The supernatants S1 and S1' were combined and further centrifuged at 20,000 g for 30 min. The resulting pellet (P2) was the crude synaptosome fraction. The pellet (P2) was washed by gently swirling it in fresh sucrose buffer whose volume was equivalent to the discarded supernatant (S2) and centrifuged again at 20,000 g for 30 min to yield the washed crude synaptosomes (P2').

### 2. Synaptosomal stimulation

As described previously, freshly prepared, washed crude synaptosomes (P2') were re-suspended in equal volumes of 2X Kreb's Ringer Phosphate (KRP) buffer whose final concentration was 10 mM Tris-HCl, 2.2 mM CaCl_2_, 0.5 mM Na_2_HPO_4_, 0.4 mM KH_2_PO_4_, 4 mM NaHCO_3_, 80 mM NaCl pH 7.5 [22]. Aliquots of the synaptosomes in KRP buffer were stimulated by the addition of increasing concentrations of KCl as indicated in the experiments and incubated at 37°C for 45 min. A non-stimulated sample served as the control. For analysis of synpatosomal lysate for immuno-blotting or radioactive GAD activity assays, the synaptosomes in each aliquot were lysed in brain lysis buffer whose final concentration was 50 mM Tris-HCl, 150 mM NaCl, 2 mM EDTA, pH 8.0, 1% Triton-X-100 and 1∶100 dilution of mammalian protease inhibitors (Sigma-Aldrich, MO, USA). The lysates were rocked at 4°C for 30 min and were centrifuged at 25,000 g for 30 min. The supernatant was collected and the protein concentrations were normalized to serve as the material for subsequent steps.

### 3. Preparation of synaptic vesicles

Synaptic vesicles were prepared as described previously [17]. All steps were conducted at 4°C. Synaptosomes (P2') were rapidly osmolysed in 10 volumes of water containing a 1∶100 dilution of mammalian protease inhibitors (Sigma-Aldrich, MO, USA). The diluted synaptosomal lysate was re-homogenized, incubated in ice for 45 min and was centrifuged at 47,000 g for 15 min to remove the large membrane fractions and mitochondria. The supernatant (S3) was further centrifuged at 200,000 g for 2 hr to yield the crude synaptic vesicles (P4). In order to entirely remove any contaminating diluted cytosolic fractions (S3), the tight pellet (P4) bearing the crude synaptic vesicles was washed thrice with 10 volumes of standard GAD buffer (50 mM potassium phosphate, 1 mM 2-aminoehtylisothiouronium bromide (AET) and 0.2 mM pyridoxal 5′-phosphate, pH 7.2), homogenized and re-suspended in the same buffer to be used for ensuing steps.

### 4. Preparation of primary rat embryo neuron enriched culture

Primary rat embryo neuron enriched cultures were prepared as described in our previous publication [57]. All dissection instruments as well as glassware used to prepare the media were autoclaved before the experiment. In the animal dissection area, Sprague Dawley timed-pregnant rats carrying E17-E18 embryos were deeply anesthetized using the inhalation anesthetic isoflurane. Rat embryos were extracted and collected in a petri dish. In a laminar flow hood, the embryos were isolated from their respective embryonic sacs and placed in Basal Medium Eagle's (Sigma-Aldrich, MO, USA) supplemented with 2 mM L-glutamine (Invitrogen, CA, USA), 5 mg/ml glucose (Invitrogen, CA, USA) and 20% heat inactivated fetal bovine serum (Invitrogen, CA, USA). This media was referred to as Growth Medium Eagles (GME). After removing the meninges, brain cortices from littermates were isolated and placed in a separate dish containing fresh GME. All cortices were pooled and mechanically dissociated using a 14-G cannula by passing them in and out of GME. The tissue suspension was centrifuged at 500 g for 5 min. The pellet was further re-suspended in GME and plated on desired culture plates pre-coated with 5 ug/ml Poly D-Lysine (PDL) (Sigma-Aldrich, MO, USA). The plates were incubated in an incubator maintained at 37°C, 5% CO_2_ and 99% humidity. After 30 min of incubation in GME, the media was replaced with Neurobasal (NB) medium supplemented with B27 (Invitrogen, CA, USA) and 0.5 mM glutamine (Invitrogen, CA, USA) which promotes selective growth of neurons while limiting glial cell growth. Cultures at 11 day *in vitro* (DIV) were used for experimentation.

### 5. KCl stimulation of primary neuronal cell cultures

Cultured neurons at 11 DIV, which show typical neuronal morphologies with healthy cell bodies and intact processes, were chosen for experiments. Fresh KCl solubilized in NB medium was used for the treatment. KCl was administered at different time points as indicated in the experiments.

### 6. Immuno-blot analysis

Protein concentrations of the samples were determined using Bradford reagent (Bio-Rad, CA, USA) with the aid of NanoDrop 8000 spectrophotometer (Thermo Scientific, DE, USA). Unequal protein concentrations were normalized with the corresponding buffer and about 20 µg of proteins per sample were electrophoretically separated on pre-cast 4–12% or 10% Bis-Tris gels (Invitrogen, CA, USA). After the run was complete, proteins were transferred onto a 0.45 µm nitrocellulose membrane (Bio-Rad, CA, USA). Post-transfer, the efficiency of the transfer was visually inspected by staining the membrane with Ponceau Stain (Sigma-Aldrich, MO, USA) followed by extensive washing with the spent transfer buffer to remove the Ponceau stain. Non-specific antibody binding was blocked by incubating the membrane in blocking buffer constituting 5% non-fat dry milk, 0.1% Tween in TBS, (TBS-T) for 1.5 hr at room temperature (RT). Subsequently, the membrane was incubated overnight at 4°C in the appropriate dilution of primary antibody (ab) prepared in TBS-T (Please refer to Table 1 for ab dilutions). All steps ensuing primary ab incubation were performed at RT. Excess primary ab was removed by washing the membrane in TBS in intervals of 30 min and two 15 min washes. Mouse or rabbit horse-radish peroxidase conjugated secondary ab (GE Healthcare Bio-Sciences Corp, NJ, USA) in TBS-T (1∶4000) was applied to the membrane and incubated for 1 hr. Following this, the membrane was further washed thoroughly in TBS before protein bands were detected by electrochemiluminescent substrate reagents (Pierce Thermo Scientific, IL, USA). The signal was captured and visualized on a highly sensitive double emulsion blue film (Midwest Scientific, MO, USA).

**Table 1:** Primary Antibody information.

Antibody	Species	Supplier	Dilution
GAD6 (epitopes directed against C terminus of GAD65 and picks up both fGAD65 and tGAD65 on IB)	Mouse monoclonal	Abcam, MA, USA.	1∶250
GAD67 (epitopes directed against 3–101 aa of GAD67 and picks up both fGAD67 and tGAD67 in IB)	Mouse monoclonal	Synaptic Systems, Goettingen, Germany.	1∶500
Calpastatin	Mouse monoclonal	Millipore Corporation, MA, USA.	1∶500
GAPDH	Rabbit monoclonal	Cell Signaling Technology, MA, USA.	1∶2000
Synaptophysin	Mouse monoclonal	Sigma-Aldrich, MO, USA.	1∶2000
VGAT	Rabbit polyclonal	Synaptic Systems, Goettingen, Germany.	1∶500
Calnexin	Rabbit polyclonal	Abcam, MA, USA.	1∶500
58K Protein	Mouse monoclonal	Abcam, MA, USA.	1∶500

Abbreviations used: GAD: Glutamic Acid Decarboxylase; GAPDH: Glyceraldehyde-3-phosphate dehydrogenase; VGAT: Vesicular GABA Transporter; IB: Immunoblotting.

### 7. Immunoprecipitation

Indirect immunoprecipitation method was employed to pull down fGAD65 as well as tGAD65 complexes from synaptosomal lysates whose synaptosomes were subjected to increasing amounts of KCl stimulation as indicated in the experiments. Unstimulated synaptosomes served as the control. For treatment of synaptosomes with calpain inhibitor, the synaptosomes were pre-incubated for 30 mins with 1 µM of calpain inhibitor peptide (Sigma-Aldrich, MO, USA) prior to KCl stimulation. All steps were performed at 4°C and with constant agitation. The protein concentrations between groups were normalized. Mouse monoclonal GAD6 ab whose epitopes are directed against the C terminus of GAD65 protein was used to pull down both fGAD65 and tGAD65. About 50 µl of Ms-GAD6 ab was added to ∼250 µg pre-cleared synpatosomal lysate and incubated overnight. In order to capture the ab coupled to the antigen (ag) in the synaptosomal lysate, about 3 mg of washed, Dynabeads Protein G (DPG) magnetic beads (Invitrogen, CA, USA) was added and the mixture was allowed to incubate for 2 hr. The DPG-ab-ag complex was isolated from the rest of the sample with the aid of DynaMag™-2 magnet (Invitrogen, CA, USA). In order to eliminate any non-specific binding on the DPG, the immunocomplex was washed at least thrice with 10 volumes of standard GAD buffer and the complex was finally re-suspended in 150 ul of the same buffer.

### 8. Cell Viability Assay

The number of viable rat primary cortical neurons at 11 DIV post 100 mM KCl stimulation for different time intervals as indicated, was assessed using the Cell Titer-Glo Luminescent Cell Viability Assay (Promega) according to the manufacturer's instructions. Briefly, equal volume of the equilibrated assay reagent was added to the neurons in a 96 well plate format and incubated for 10 min at RT. The homogeneous “add-mix-measure” format resulted in cell lysis and generation of a luminescent signal proportional to the amount of ATP present, which is an indicator of metabolically active cells. After transferring the lysate to a standard opaque walled multi-well plate, the intensity of the luminescent signal was measured with a luminometer (SpectraMax, Molecular Devices, CA, USA).

### 9. Semi-quantitative RT-PCR

After KCl stimulation, the cell culture media from the tissue culture plates was aspirated and discarded. Total RNA enriched in mRNA from the KCl stimulated cells was extracted using the RNeasy Mini Kit (Qiagen, CA, USA) according to the manufacturer's directions. The RNA concentrations were evaluated using the NanoDrop 8000 spectrophotometer (Thermo Scientific, DE, USA) and normalized prior to using as a starting material for c DNA synthesis. c DNA was synthesized using the Superscript® III First- Strand Synthesis SuperMix (Invitrogen, CA, USA) according to the manufacturer's instructions. Briefly, up to 5 µg of total RNA was incubated with 50 µM of oligo(dT)_20_ at 65°C for 5 min. Reverse transcription reaction was carried out at 50°C for 50 min with a supplied 10 µl of 2X First-Strand Reaction Mix and 2 µl of Superscript® III/RNaseOUT™ Enzyme Mix. Amplification of the prepared c DNA was performed using the Platinum PCR Supermix (Invitrogen, CA, USA) in mastercycler gradient thermal cycler (Eppendorf, NY, USA). The reaction mixture (50 µl) consisted of 45 µl of PCR pre-mix consisting of 2 U complexed *Taq*DNA polymerase with Platinum *Taq* Antibody, 2.2 mM Tris-HCl (pH 8.4), 5.5 mM KCl, 0.165 mM MgCl_2_, 2.2 µM each of dNTPs and 200 nM per primer; 1 µl each of primers and 3 µl template DNA solution. The sequences of the primers chosen to study the expression of GAD65 and GAD67 [58] and the control G3PDH [59] mRNAare shown in Table 2. The amplification conditions consisted of brief priming at 94°C for 2 min followed by 35 cycles of denaturation at 94°C for 30 secs, annealing at 54°C for 30 secs and an extension step of 72°C for 1 min and a final single long extension of 72°C for 10 min. The PCR products were separated on a 1.5% agarose gels. Gels were stained with Ethidium bromide, illuminated on a UV transilluminator, and documented on a black-and-white instant film.

**Table 2:** Sequence of primers used in the RT-PCR reaction.

Gene Name	Primer Sequence (5′-3′)	PCR Product Length (bp)
GAD65	F: GGCTCTGGCTTTTGGTCCTTCR: TGCCAATTCCCAATTATACTCTTGA	441
GAD67	F: GCTGGAAGGCATGGAAGGTTTTAR: AATATCCCATCACCATCTTTATTTGACC	303
G3PDH	F: TCCATGACAACTTTGGCATCGTGGR: GTTGCTGTTGAAGTCACAGGAGAC	366

Abbreviations used: G3PDH: Glyceraldehyde-3-phosphate dehydrogenase.

### 10. Radioactive GAD activity assay

GAD activity was assayed by a classical radiometric method measuring the formation of ^14^CO_2_ liberated from end labeled [L-^14^C] glutamic acid as described [25]–[26]. Briefly, the reaction set up consisted of test tubes containing aliquots of GAD in standard GAD buffer, to which 0.25 µCi/ml end labeled [L-^14^C] glutamic acid (Perkin Elmer, MA, USA) was added. The total reaction mixture in each test tube was 200 ul. As soon as the hot substrate was added, 0.6 ml eppendorf tube containing 200 ul benzethonium hydroxide (BH) (Sigma-Aldrich, MO, USA) was suspended half-way through the test tube. The test tubes were tightly sealed with a rubber stopper and were subjected to constant shaking for 45 min at RT. The reaction was stopped by injecting 200 ul of 0.5 N H_2_SO_4_ through the rubber stopper directly into the GAD mixture without disturbing the tube containing BH. The experimental set up facilitated the capture of ^14^CO_2_ gas by BH, released in the decarboxylation reaction of end labeled [L-^14^C] glutamic acid to GABA, which is directly proportional to the activity of the GAD enzyme. Thereafter, the test tubes continued to shake overnight, until all of the ^14^CO_2_ was captured by BH. The radioactivity in the BH was measured by suspending the eppendorf tubes in 5 ml of Econo- Safe™ scintillation cocktail (Research Products International Corporation, IL, USA) contained in scintillation vials and the emissions were recorded using the Tri-Carb 2900TR Liquid Scintillation Analyzer (Perkin Elmer, MA, USA).

### 11. *In vitro* calpain cleavage assay

The assay was conducted in a similar method as described previously [22]. Washed, enriched SVs were prepared as mentioned above (Refer to preparation of synaptic vesicles). SVs were suspended in a calpain reaction buffer (10 mM HEPES-NaOH, pH 7.5, 150 mM NaCl, 1 mM EGTA) and homogenized manually using a pencil Teflon homogenizer. Increasing amounts of the human calpain-1 enzyme (Sigma-Aldrich, MO, USA) accompanied by 10 mM CaCl_2_ was added to the aliquots of SVs except for the internal control. The calpain reaction was performed at 30°C for 45 min. After the stipulated time, SVs were diluted 10X and were centrifuged at 200,000 g for 1.5 hr. The aqueous fraction was collected and was concentrated using Centricon centrifuge concentrator (Millipore, MA, USA). The remainder of tight SV membrane pellets were gently washed thrice with 10 volumes of calpain reaction buffer. The SV membranes were then re-suspended in calpain reaction buffer. The concentrated aqueous fraction and the homogenized SV membranes were normalized, resolved on 10% Bis-Tris gels (Invitrogen, CA, USA) and analyzed by immuno-blotting against GAD6 ab.

### 12. Vesicular uptake assay of GABA newly synthesized from [L-^3^H] glutamate

Newly synthesized GABA uptake from [L-^3^H] glutamic acid was conducted as described previously in a detailed manner [17], [60]–[61]. Briefly, SVs were isolated as described in the section above. After washing the SVs of contaminating cytosolic fractions with GPBS buffer (9.5 mM KH_2_PO_4_, 40.5 mM K_2_HPO_4_, 8 mM KCl, 86.6 mM potassium gluconate, pH 7.4), SVs (2 mg/ml) were equally distributed into 2 aliquots. 10 mM CaCl_2_ was added to both the tubes to provide calcium for the calpain reaction in the next step. One of the aliquots received treatment with 0.15 U calpain at 30°C for 45 min, while the other served as a control. After calpain treatment, both the control and calpain-treated SVs were washed thrice with GPBS buffer to serve as a starting material for the uptake assay.

### 13. Creation of transient focal cerebral ischemia/reperfusion stroke rat model

Male adult Sprague-Dawley rats (weighing 250–300 g) were subjected to middle cerebral artery occlusion (MCAO) for 90 min using the intra-luminal thread occlusion method as described [28]. All animals were fasted the night before the procedure. On the day of occlusion, rats were anaesthetized initially with isoflurane, and then during surgery ketamine (80 mg/kg) and xylazine (4 mg/kg) were administered via intraperitoneal injection. The body temperatures of the animals were maintained at 37.5°C throughout the surgery using a heating lamp and a heating pad connected to rectal probe. In all experiments, the left side of the brain was chosen to be the ischemic side while the matching right side was chosen to be the control region. A laser doppler probe attached to a laser doppler flow monitor (Perimed Inc, OH, USA) was positioned between the bregma and the lamda on the ipsi-lateral side to monitor the cerebral blood flow (CBF). Silicon-coated 4.0 nylon monofilament suture was advanced 18–22 mm from the external carotid artery bifurcation into the internal carotid artery until there was a slight resistance.

### 14. Identification of the injured tissue by TTC staining

The MCAO rat whole brain was harvested immediately after 90 min of occlusion minimizing tissue rupture as much as possible. The whole brain was then snap-frozen at −80°C for 7 min to harden the tissue. Thereafter, at RT, the whole brain was positioned in a rat brain matrix (Zivic Instruments, PA, USA). Blades were placed on it to incise the brain to obtain 2 mm coronal brain sections. The sections were immediately immersed in 1% 2, 3, 5-triphenyl tetrazolium chloride (TTC) in PBS for 5 min at 37°C for the reaction to develop. Injured tissue stained white, while the uninjured tissue stayed pink. Subsequently, after visual inspection, the core and the penumbra of the ischemic side and the matching control side were identified and dissected out. The brain tissues were then processed with lysis buffer as mentioned in the previous sections [see synaptosomal stimulation], to obtain their respective lysates to serve as a starting material for immuno-blot analysis.

### 15. Glutamate microdialysis

For those animals subjected to microdialysis for measuring glutamate release, guide cannulae were pre-implanted at least 1 week before microdialysis and MCA occlusion. Briefly, rats were anaesthetized with a combination of xylazine (4 mg/kg, i.p.) and ketamine (80 mg/kg, i.p.) and then placed on a stereotaxic frame (Stoelting Co., IL, USA). The skull was surgically exposed for implantation of a sterile 22-gauge guide cannula at stereotaxic coordinates for two striatal regions: 1) 0.5 mm anterior to bregma, 3.0 mm lateral to the midline and 2.0 mm ventral to the skull and 2) 2.5 mm posterior to bregma, 4.0 mm lateral to midline and 2.0 mm ventral to the skull. The guide cannulae were then secured into place with acrylic dental cement and skull screws. One day before microdialysis and MCA occlusion, a hollow probe was inserted through the cannula into each of the two striatal regions for glutamate microdialysis. The coordinates for the tip of the probe were 0.5 mm anterior to bregma, 3.0 mm lateral to the midline and 7.0 mm below the skull and 2.5 mm posterior to bregma, 4.0 mm lateral to midline and 7.0 mm below the skull. After probe insertion, rats were placed in a chamber attached to a fluid swivel that allowed animals to move freely (Raturn® system; Bioanalytical System Inc., IN, USA). The dialysis probes were perfused overnight with KRP buffer at a rate of 1.0 µl/min.

The next day, after two baseline samples, animals were subjected to 90-min MCAO. Dialysis samples were collected at a 30 min interval before and during MCAO and analyzed by high performance liquid chromatography (HPLC) – electrochemical detection (HTEC-500; Eicom, Kyoto, Japan). Separation of glutamate was achieved on a 150 mm×1 mm i.d. column packed with a TSK gel ODS-80 TM, 5 µm particle size. The potential on the graphite working electrode was set at +400 mV (relative to the Ag/AgCl reference electrode). The mobile phase was made of 0.1 M phosphate buffer (pH 6.0), 4% methanol, and 40 mg/l EDTA and pumped at a rate of 500 µl/min.
